# Delayed Diagnosis of Perimandibular Abscess in a Woman With Social Isolation: A Case Report

**DOI:** 10.7759/cureus.94505

**Published:** 2025-10-13

**Authors:** Hidehiro Someko, Makoto Adachi

**Affiliations:** 1 Department of Healthcare Epidemiology, Kyoto University, Kyoto, JPN; 2 Department of Systematic Reviewers, Scientific Research WorkS Peer Support Group (SRWS-PSG), Osaka, JPN; 3 Department of Internal Medicine, Nagoya Tokushukai General Hospital, Kasugai, JPN; 4 Department of Oral and Maxillofacial Surgery, Nagoya Tokushukai General Hospital, Kasugai, JPN

**Keywords:** anaerobe, chronic kidney disease of unknown aetiology, diagnostic delay, loneliness in medicine, oral infection

## Abstract

Social isolation is associated with increased mortality and adverse health outcomes, though its relationship with healthcare utilization remains complex. We present a case of a socially isolated woman in her 70s whose delayed medical consultation contributed to the progression of an initially treatable mandibular infection to a fatal perimandibular abscess. Approximately three months before admission, she began experiencing dental pain but did not seek treatment, in the context of poor oral hygiene and social isolation. A community welfare volunteer eventually found her in a markedly deteriorated state, but she refused transport for one week before agreeing to seek care. She presented in septic shock with altered mental status and left facial swelling. Laboratory studies revealed severe systemic inflammation, and imaging identified an extensive left perimandibular abscess. Blood cultures grew *Parvimonas micra*, an anaerobic oral commensal. Despite antimicrobial therapy and drainage, infection control was not achieved due to the extent of the abscess, and she died on hospital day 32. This case illustrates how poor oral hygiene and social isolation can together contribute to delayed diagnosis and fatal infectious complications, underscoring the need for proactive symptom screening among socially vulnerable older adults.

## Introduction

Social isolation affects approximately 20% of adults and is increasingly recognized as a major public health concern, particularly among older populations [1,2]. It has been associated with increased risks of mortality and dementia [3,4]. While some studies suggest that social isolation may lead to delayed access to medical care, the evidence remains inconclusive, with most reports limited to cross-sectional associations rather than documenting specific clinical trajectories [5-9].

Perimandibular abscess secondary to odontogenic infection can be a serious deep neck infection. Predisposing factors include poor oral hygiene, dental infections, dehydration, reduced salivary flow, and immunosuppression [10]. Current treatment approaches vary widely and may include antibiotics alone, aspiration with or without imaging guidance, or incision and drainage, though evidence-based treatment guidelines remain limited [11].

Oral health carries particular significance in older populations. Age-related reductions in salivary flow, medication effects, and barriers to dental access predispose individuals to periodontal disease and dental caries, which may progress from localized infection to deep neck space involvement [12]. Beyond local complications, untreated dental infections are associated with systemic adverse outcomes--bacteremia, sepsis, endocarditis in at-risk patients, and aspiration pneumonia--and may exacerbate comorbidities such as diabetes and chronic kidney disease.

We present a case of a socially isolated older woman whose delayed medical consultation contributed to the progression of an initially treatable mandibular infection to a fatal perimandibular abscess. This case provides a rare documented clinical timeline illustrating how social isolation translates into delayed healthcare-seeking behavior and ultimately fatal progression of a treatable infection, thereby complementing the existing literature's gap between suggested associations and quantitative evidence.

## Case presentation

A woman in her 70s was brought to the emergency department via ambulance after a community welfare volunteer, who had been monitoring her through a municipal program, found her in a markedly deteriorated state and unable to move. Although the volunteer had first discovered her in this condition a week earlier and had called for emergency assistance at that time, the patient had firmly refused transport. Daily follow-up visits ensued, during which she continued to decline care. On the day of admission, she finally agreed to seek medical attention, but shortly after expressing willingness, her consciousness began to decline, prompting a second emergency call and her eventual transport.

Three months prior to admission, she had begun experiencing dental pain but had not sought treatment throughout this period. She did not use any medications or home remedies to manage the pain. She rarely brushed her teeth and had no history of regular oral hygiene practices. She lived alone, had no children, was widowed 25 years earlier, and had lost contact with her older brother, who remained unreachable despite repeated efforts by the volunteer.

Her medical history was significant for moderate aortic stenosis, severe mitral regurgitation, hypertension, chronic kidney disease, and dyslipidemia. She had been under regular cardiology follow-up, with transcatheter aortic valve implantation (TAVI) recommended but not yet performed. Her medications included a telmisartan/amlodipine combination, azosemide 30 mg, rosuvastatin 2.5 mg, amlodipine 5 mg, ferrous citrate 50 mg, indapamide 1 mg, calcium polystyrene sulfonate 25 g (all daily), and darbepoetin alfa 4 mcg twice weekly.

**Figure 1:**
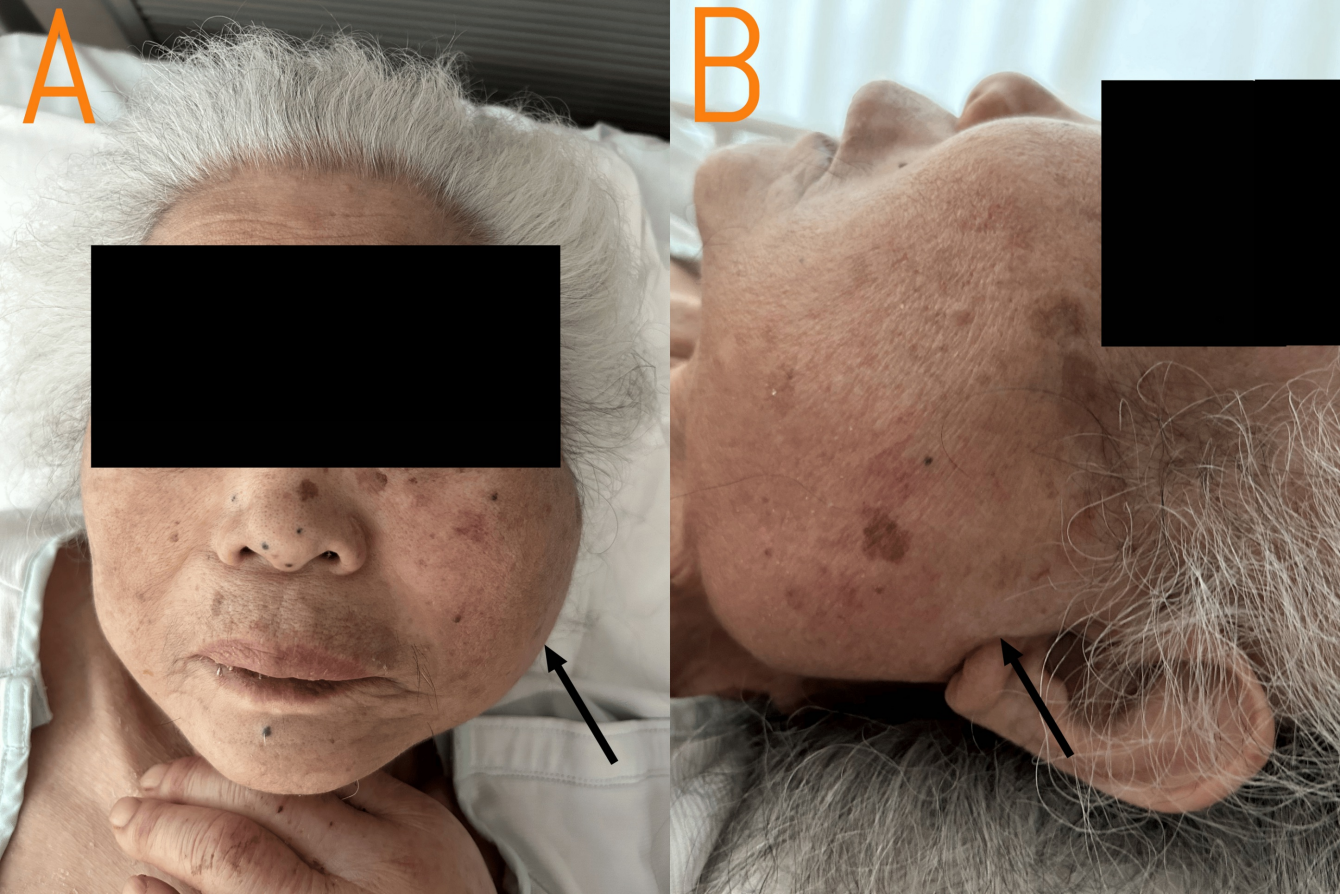
Facial swelling on hospital day 8 (A) Frontal view showing facial edema with asymmetric involvement. (B) Left lateral view demonstrating prominent left parotid-masseteric region swelling. The swelling was firm on palpation, initially raising a differential diagnosis including a parotid gland tumor. Photography from admission was not available; this image represents the progression of facial swelling by hospital day 8, following initial presentation with firm swelling in the left parotid-masseteric region.

On arrival, she presented with altered mental status (Glasgow Coma Scale E3V4M6), hypothermia (34.9°C), blood pressure 102/74 mmHg, tachycardia (135/min), tachypnea (22/min), and hypoxemia (oxygen saturation 94% on 10 L/min oxygen via reservoir mask). Physical examination was notable for a systolic ejection murmur consistent with her known valvular disease. Head and neck examination revealed bilateral facial swelling, with the left side more prominent than the right. The left parotid-masseteric region showed firm swelling on palpation, initially raising differential diagnoses, including parotid gland tumor, before imaging studies confirmed the infectious etiology. There was no reported lymphadenopathy, thyroid enlargement, or pharyngeal erythema. Figure 1 shows the progression of facial swelling by hospital day 8, as photography at admission was not available. Initial assessment revealed findings consistent with sepsis.

Laboratory studies revealed severe systemic inflammation with a C-reactive protein of 23.35 mg/dL and leukocytosis with left shift (white blood cell 22,000/μL with 97.2% neutrophils). She had acute-on-chronic kidney injury with serum creatinine 4.05 mg/dL (baseline approximately 1.5 mg/dL) and blood urea nitrogen 135.8 mg/dL. Blood gas analysis showed metabolic acidosis with respiratory compensation (Table 1).

**Table 1:** Laboratory data on admission Abbreviations: AST, aspartate aminotransferase; ALT, alanine aminotransferase; LDH, lactate dehydrogenase; ALP, alkaline phosphatase; BNP, B-type natriuretic peptide

Parameter	Value	Normal Range	Unit
Hematology			
White blood cell count	22	4.0-10.0	×10³/μL
Neutrophils	97.2	50-70	%
Lymphocytes	1.2	20-40	%
Red blood cell count	4.41	4.0-5.5	×10⁶/μL
Hemoglobin	12.9	12.0-16.0	g/dL
Hematocrit	39.6	36-48	%
Platelet count	162	150-400	×10³/μL
Chemistry			
Blood urea nitrogen	135.8	Aug-20	mg/dL
Creatinine	4.05	0.6-1.2	mg/dL
Uric acid	10.4	2.5-7.0	mg/dL
Sodium	139	135-145	mmol/L
Potassium	4.8	3.5-5.0	mmol/L
Chloride	102	98-108	mmol/L
Total protein	5.8	6.5-8.3	g/dL
Albumin	2.5	3.5-5.0	g/dL
Total bilirubin	0.4	0.3-1.2	mg/dL
AST	17	Oct-40	U/L
ALT	14	May-35	U/L
LDH	212	120-245	U/L
ALP	85	115-359	U/L
Amylase	33	25-125	U/L
Glucose	102	70-110	mg/dL
Inflammatory markers			
C-reactive protein	23.35	<0.3	mg/dL
Cardiac markers			
BNP	613.6	<100	pg/mL
Blood gas analysis			
pH	7.308	7.35-7.45	
PCO₂	33.7	35-45	mmHg
PO₂	47.3	80-100	mmHg
HCO₃⁻	16.9	22-28	mmol/L
Lactate	2.9	0.5-2.2	mmol/L

**Figure 2:**
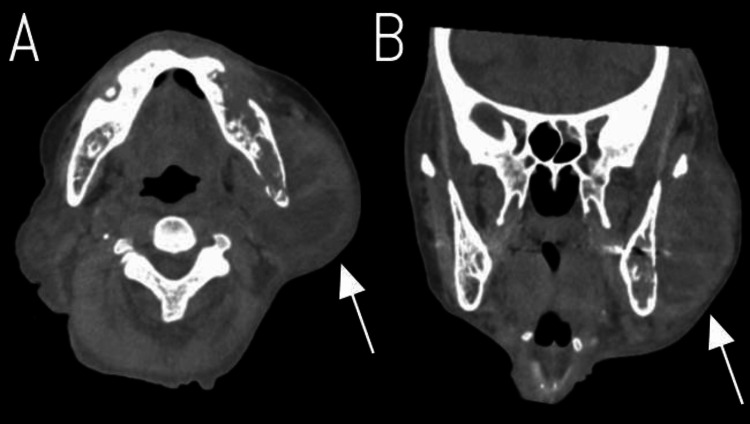
Head computed tomography on hospital day 7 revealing a left perimandibular abscess (A) Axial view and (B) coronal view demonstrating a large, well-defined fluid collection in the left perimandibular region (white arrows). The abscess shows characteristic rim enhancement and causes mass effect with compression of surrounding structures.

Blood cultures were obtained, and empiric antibiotic therapy with piperacillin/tazobactam 4.5 g every 12 hours was initiated for presumed septic shock. On hospital day 4, blood cultures grew *Parvimonas micra*, an anaerobic oral commensal, suggesting an oral or dental source. Antibiotic therapy was subsequently changed to ampicillin based on the culture results and sensitivity pattern. Head computed tomography revealed an extensive left perimandibular abscess on hospital day 7 (Figure 2).

**Figure 3:**
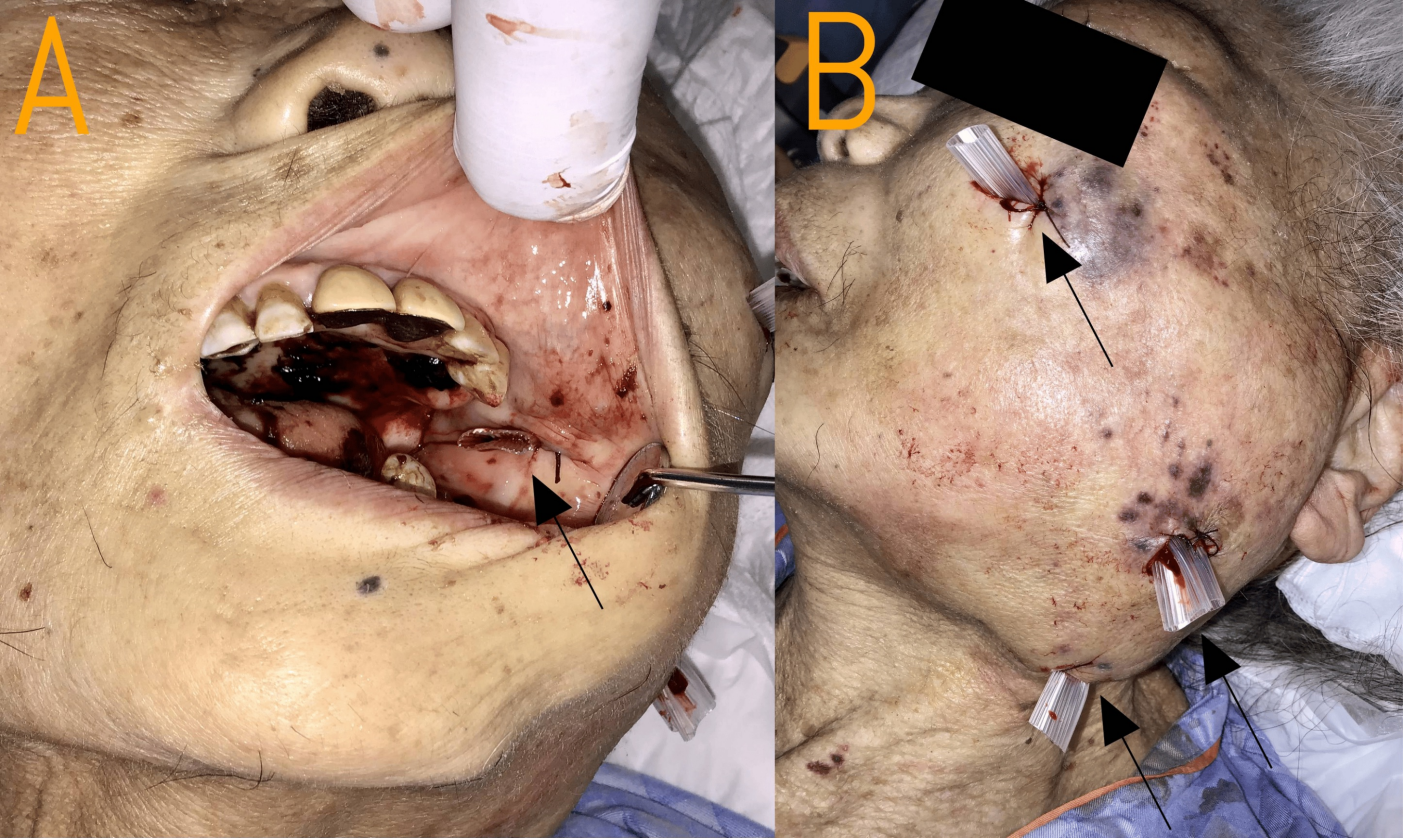
Abscess drainage procedure on hospital day 17 (A) Intra-oral view showing drain placement (black arrow) from the anterior border of the mandibular ramus to the medial aspect of the mandibular ramus for internal drainage of the perimandibular abscess. (B) External view demonstrating multiple drains (black arrows) placed in the left cheek, left parotid-masseteric region, and left submandibular area for comprehensive drainage of the extensive abscess cavity. Despite multiple drain placement, purulent drainage persisted, indicating the need for more extensive surgical debridement that was ultimately not performed due to the patient's poor overall condition and treatment preferences.

Oral surgery consultation was obtained and extensive drainage under local anesthesia was performed on hospital day 17, with drain placement in multiple sites, including the left cheek, left parotid-masseteric region, and left submandibular area externally, as well as intraorally from the anterior border of the mandibular ramus to the medial aspect of the mandibular ramus (Figure 3).

Despite drain placement, purulent drainage persisted, requiring additional drain insertion. However, the abscess was too extensive for adequate drainage through limited incisions, and more extensive surgical debridement was recommended. Given her deteriorating systemic condition, with inflammatory markers remaining elevated, renal function continuing to worsen (serum creatinine remaining above 4.0 mg/dL), increasing somnolence, minimal oral intake likely reflecting a uremic state, and her consistent refusal of hemodialysis, along with her verbally expressed preferences regarding invasive interventions, the decision was made to pursue conservative management rather than proceed with extensive surgical debridement.

Despite continued antimicrobial therapy and palliative abscess drainage, infection control was not achieved, and she passed away on hospital day 32.

## Discussion

This case illustrates the delayed diagnosis of a perimandibular abscess in a socially isolated older woman, ultimately resulting in sepsis and death. The patient had experienced untreated dental pain for months, had poor oral hygiene practices, and did not seek medical or dental care. This case highlights two important clinical lessons: first, poor oral hygiene can lead to severe and potentially fatal infections, such as perimandibular abscess and sepsis; second, social isolation may hinder timely symptom reporting and medical access, underscoring the need for proactive screening among socially vulnerable older adults.

Poor oral hygiene can progress to fatal infections, including a perimandibular abscess. While a perimandibular abscess is not uncommon in clinical practice, progression to fatal systemic complications, as seen in this case, is relatively rare. A perimandibular abscess is a serious deep neck infection, often resulting from odontogenic infection [13]. Predisposing factors include poor oral hygiene, dental infections, dehydration, reduced salivary flow, and immunosuppression [10]. In this case, the detection of *Parvimonas micra*, an anaerobic oral commensal, strongly suggested an oral source. Older adults may be especially susceptible due to age-related decline in salivary function, impaired self-care, and limited access to dental services. Moreover, in this patient, chronic kidney disease may have contributed to impaired immune defense, potentially facilitating the spread of infection. This case underscores the need for maintaining oral health in older individuals, as even common dental neglect may result in life-threatening complications.

This case also highlights how social isolation may contribute to delayed care by reducing both symptom recognition and help-seeking behavior. Despite experiencing persistent dental pain for several months, the patient did not seek medical or dental attention, nor did she share her symptoms with others, including a community welfare volunteer or her cardiologist, with whom she had ongoing follow-up. Although the literature on the direct relationship between social isolation and delayed care remains inconclusive [5-9], both conditions may share underlying factors such as low health literacy, reluctance to seek help, and limited trust in healthcare systems. These factors may collectively contribute to delayed diagnosis and treatment among socially isolated individuals. Passive approaches that rely on patient-initiated complaints may be insufficient in such populations. Instead, structured and proactive symptom screening should be incorporated into regular contact opportunities, such as home visits, community outreach programs, or routine primary care encounters, particularly to detect subtle but potentially serious conditions before they progress to life-threatening complications, as occurred in this case.

This case also prompts reflection on potential opportunities for earlier intervention within existing healthcare touchpoints. The patient maintained regular follow-up with her cardiologist, which could have served as a point of contact for broader health assessment. While specialists understandably focus on their domain of expertise, this case suggests potential value in brief screening questions about general well-being, oral health, or changes in daily functioning during routine visits, particularly for older adults living alone. Such an approach need not burden specialists with additional clinical responsibilities, but rather could serve as a gateway for appropriate referral or connection with primary care or social services.

## Conclusions

This case illustrates how poor oral hygiene, when combined with social isolation, can contribute to delayed diagnosis and fatal infectious complications such as perimandibular abscess and sepsis. Routine oral health assessment and proactive symptom screening should be considered essential components of preventive care for older adults, particularly those experiencing social isolation. Healthcare providers and community-based programs should recognize social isolation as a potential barrier to timely diagnosis and adopt structured strategies to detect early signs of deterioration before critical illness occurs.
